# The Prevalence and Predictors of Atherosclerotic Coronary Artery Disease in Rheumatic and Non-rheumatic Valvular Heart Disease Patients

**DOI:** 10.7759/cureus.57317

**Published:** 2024-03-31

**Authors:** Shahida Shafi, Sihem Aouabdi, Ziad A Taher, Abdulrahman E Alghamdi, Mohammed A Ahmed, Fatima A Ahmed, Suliman Alghamdi, Ali Haneef

**Affiliations:** 1 Department of Cardiology, King Abdullah International Medical Research Center, King Saud Bin Abdulaziz University for Health Sciences, Jeddah, SAU; 2 Department of Regenerative Medicine, King Abdullah International Medical Research Center, King Saud Bin Abdulaziz University for Health Sciences, Jeddah, SAU; 3 Department of Medicine, King Abdullah International Medical Research Center, King Saud Bin Abdulaziz University for Health Sciences, Jeddah, SAU; 4 Department of Medicine, Ministry of National Guard Health Affairs, King Abdulaziz Medical City, Jeddah, SAU; 5 Department of Psychiatry, University of Toronto, Toronto, CAN; 6 Department of Emergency Medicine, King Abdullah International Medical Research Center, King Saud Bin Abdulaziz University for Health Sciences, Jeddah, SAU; 7 Department of Cardiology, Ministry of National Guard Health Affairs, King Abdulaziz Medical City, Jeddah, SAU; 8 Department of Radiation Oncology, Ministry of National Guard Health Affairs, King Abdulaziz Medical City, Jeddah, SAU; 9 Department of Cardiac Surgery, King Faisal Cardiac Center, King Abdulaziz Medical City, Jeddah, SAU

**Keywords:** risk factors, valve lesions, predictors, valvular heart disease, coronary artery disease

## Abstract

Objectives: The paradox of concurrent coronary artery disease (CAD) among patients with rheumatic and non-rheumatic valvular heart disease (RVHD; non-RVHD) is unclear. We aimed to evaluate the impact of the RVHD and non-RVHD on the prevalence of CAD and various risk factors, assess the number of diseased coronaries, clinical profile and the possible predictors of CAD in these patients, which may clarify the paradox and provide an insight for the prevention of CAD.

Methods: The records of 106 valvular heart disease patients who had undergone valve replacement surgery at the King Faisal Cardiac Centre from January 2014 to October 2019 were evaluated. The clinical data and established risk factors were compared and logistic regression analyses were performed to identify plausible predictors of CAD.

Results: Transthoracic echocardiographic diagnosis of 106 patients confirmed, 43 had RVHD (56.4 ± 8 years), of whom six (13.9%) had CAD with the highest mitral valve regurgitation (*p* < 0.01), and 63 had non-RVHD (60.0 ± 12 years). Of these, 31 patients showed the highest CAD (49.2%). Single- and triple-vessel disease was most common in RVHD and non-RVHD patients with concurrent CAD (33.3%; 41.9%, respectively), while non-RVHD patients also had quadruple vessel disease. The mean age of the RVHD and non-RVHD patients with coexisting CAD was significantly higher (66.7 ± 5; 66.7 ± 8 years) than those without CAD (46.1 ± 12.0; 54.7 ± 20, respectively). RVHD patients showed a significantly lower prevalence of diabetes, dyslipidaemia, hypertension, inflammatory cells, hepatorenal function markers, ejection fraction, and regional wall motion abnormality compared to RVHD patients with coexisting CAD (p < 0.01). Bivariate analysis indicated white blood cells, monocytes, neutrophils, gamma-glutamyl-transferase (GGT), bilirubin and blood urea nitrogen (BUN) to be significantly lower in RVHD patients. Predictors of high risk of CAD were BUN and hyperlipidaemia for RVHD and BUN, creatinine and GGT for non-RVHD patients.

Conclusions: The prevalence of CAD in Saudi RVHD patients was significantly lower than in the Western countries, whereas non-RVHD was higher. The low prevalence may partly be attributed to age, reduced mitral regurgitation, and low frequency of risk and inflammatory factors.

## Introduction

Coronary artery disease (CAD) and valvular heart disease (VHD) are major global cardiovascular disorders that seriously impact healthcare systems and economics. In 2015, the World Health Organization (WHO) reported the prevalence of rheumatic valvular heart disease (RVHD) to be 33.4 million cases with 305,000 deaths occurring annually, and this trend in mortality is expected to increase [1]. In Saudi Arabia, the incidence in 2010 was 24 per 10,000 children (5-14 years), still higher than the global rate [2]. Indeed, atherosclerotic CAD alone is the single most significant leading cause of mortality globally, including in Saudi Arabia, and a national survey reported a prevalence of 5.5% [3], higher than India (3%), China (2%) and Europe (5%) [4-6].

VHD comprises chronic RVHD and non-RVHD of different aetiologies and frequently presents as stenosis and regurgitation. Clinical complications of both cardiac entities are manifested as damaged valves, heart failure, stroke, and atrial fibrillation [7]. RVHD is a long-term outcome of rheumatic fever (a complication of single or multiple episodes of group A streptococcal infection), that leads to valve damage due to autoimmune and inflammatory reactions [8]. Non-RVHD is a largely degenerative disease that increases with age. The principal underlying cause of CAD is atherosclerosis, which is a chronic inflammatory disease with autoimmune components. Several risk factors and physiological pathways associated with atherosclerosis in the early stages of CAD are also common to VHD as well as genetic factors, inflammation, autoimmunity, and oxidative stress [9], but age and unmodifiable factors remain the main contributors to CAD.

The impact of chronic VHD on CAD is not well established. Limited data published from Western and developing countries show disparities in the prevalence of coexistent CAD among RVHD and non-RVHD patients and the data are still somewhat conflicting [10,11]. For instance, the overall prevalence of CAD with established RVHD and non-RVHD varied widely from 1.75% to 20% [12-16] and from 25-40% [10,16-18] respectively.

This is the first study on the atherosclerotic CAD paradox among RVHD and non-RVHD Saudi patients. In this context, we aimed to evaluate (i) the prevalence of coexistent CAD; (ii) the number of affected coronaries, and the pattern of valvular lesions; (iii) the potential risk factors in RVHD and non-RVHD patients that might be contributing to this paradox; (iv) and finally the plausible predictors of CAD.

## Materials and methods

Patients and methods

Study Patients, Procedures, and Design

In this retrospective single-center study, the records of 106 Saudi patients (age ≥ 46 years) who underwent valve replacement surgery due to rheumatic involvement at the King Faisal Cardiac Centre (The Ministry of National Guard Health Affairs Hospital) between January 2014 and October 2019 were retrieved for evaluation. Valvular lesions were confirmed as rheumatic based on transesophageal or transthoracic echocardiography and clinical diagnosis. Preoperative, coronary arteriography performed on the VHD patients confirmed a significant atherosclerotic CAD where one or more coronaries had ≥ 50% luminal narrowing. According to the diagnosis, 43 patients had confirmed RVHD (56.4 ± 8 years) and 63 had non-RVHD (60.0 ± 12 years). These two groups of patients who had significant CAD were compared with patients without CAD in terms of risk factors, valvular lesions and the number of coronaries diseased.

The study was approved by the King Abdullah International Medical Research Centre (KAIMRC), and written consent was obtained from the hospital’s Institutional Ethics Review Board (SP17-082-J-IRB) to allow targeted data retrieval. The study’s inclusion criteria were patients ≥ 40 years of age. The exclusion criteria included patients who had already undergone redo valvular surgery. It was difficult to find a similar age-matched patient from a single center and the fact that the study was terminated at the beginning of 2020 due to the start of the COVID-19 era.

Measurements

Routine preoperative biochemical (26 markers), and clinical data including demographic information, such as age, gender, body mass index (BMI) (25-30 kg/m^2^ = overweight > 30kg/m^2^ = obese), history of cardiovascular symptoms such as shortness of breath/chest pain, preoperative chest X-ray (CXR) findings, and various risk factors were noted. Preoperative results of an electrocardiogram (ECG), and coronary CT angiography were recorded. Echocardiographic examination data confirming the presence of valve regurgitation, stenosis, the number of valves affected and the severity of valvular lesion as well as the ejection fraction and regional wall motion abnormalities were retrieved.

Statistical Analysis

All collected data were analyzed by using the Statistical Package for the Social Sciences (IBM SPSS Statistics for Windows, IBM Corp., Version 23.0, Armonk, NY). In some cases, determinants ≤ 5% of the values were missing. Quantitative variables were presented as means ± SDs. The baseline characteristics of RVHD and non-RVHD patients were compared with those with concurrent CAD using the student’s t-test. Categorical data were analyzed with Fisher’s exact test (n < 5) or Pearson’s chi-square test and documented as number (n), rate (%) or odds ratio (OR) with confidence intervals (CI). Bivariate logistic regression modelling was used to pinpoint the predictors of CAD in VHD patients. Multivariate logistic regression analysis was used to support the outcome risk predictors. A p-value of ≤ 0.05 was considered statistically significant.

## Results

The prevalence of CAD in RVHD patients was 13.9% (18.8% in males and 11.1% in females), and in non-RVHD patients was 49.2% (53.5% in males and 40.0% in females). The mean age of the RVHD and non-RVHD patients with coexisting CAD was 66.7 ± 5 years and 66.7 ± 8, respectively as shown in Table 1. The gender distribution within RVHD patients with CAD was equal (50%), whereas, in the non-RVHD, the majority were predominantly males (74.1%) (Table 1). Overall, significant CAD was detected in 34.9% (n=37) of all VHD patients (70.2% male and 29.7% female). The BMI indicated that VHD patients with concurrent CAD were obese and overweight (83.3% and 67.7%, respectively).

**Table 1:** Demographics of valvular heart disease patients with/without coexisting coronary artery disease and comparison of risk factors with their relative group Fisher’s Exact (value <5) and Person Chi-square tests for risk factor comparison were used between RVHD with CAD vs. RVHD; and non-RVHD with CAD vs. non-RVHD. Significance of differences: RVHD with CAD vs. RVHD; Non-RVHD with CAD vs. Non-RVHD. ***P ≤ 0.01, **P ≤ 0.015. n, represents the number of patients. RVHD: rheumatic valvular heart disease; non-RVHD: non-rheumatic valvular heart disease; CAD: coronary artery disease. BMI: body mass index

Patients group	RVHD with CAD	RVHD	Non-RVHD with CAD	Non-RVHD
Total cases (n)	(n=6)	(n=37)	(n=31)	(n=32)
Gender, n (%)				
Male	3 (50.0)	13 (35.1)	23 (74.1)	20 (62.5)
Female	3 (50.0)	24 (64.9)	8 (25.8)	12 (37.5)
Age (years): Mean ± SD				
Male	67.3 ± 4.6	46.3 ± 12.1	67.2 ± 9.0	57.8 ± 17.5
Female	66.0 ± 4.6	46.0 ± 9.1	65.2 ± 6.2	49.6 ± 15.2
Overall	66.7 ± 5.0	46.1 ± 12.2	66.7 ± 8.4	54.7 ± 20.0
BMI (kg/m^2^), n (%)				
Normal	1 (16.7)	10 (27.0)	10 (32.0)	11 (34.0)
Obese & overweight	5 (83.3)	27 (73.0)	21 (67.7)	20 (62.5)
Risk factors: n, (%)				
Diabetes	6 (100)	4 (10.8) ***	21 (67.7)	10 (31.3) **
Dyslipidaemia	5 (83.3)	11 (29.7) ***	17 (54.8)	8 (25.0) **
Hypertension	5 (83.3)	10 (27.0) **	24 (77.4)	15 (46.9) **
Obesity	2 (33.3)	11 (29.7)	9 (29.0)	10 (31.3)
Smoking	1 (16.7)	3 (8.1)	2 (6.5)	1 (3.1)

A comparison of risk factors for CAD in Table 1 showed that although the major risk factors were common among all patients; the CAD patients with the two cardiac valvular entities had significantly increased prevalence of diabetes, dyslipidemia, and hypertension (0.001 < p < 0.015).

Vessel and valve analysis, the severity of disorders, and cardiac wall abnormalities

**Figure 1:**
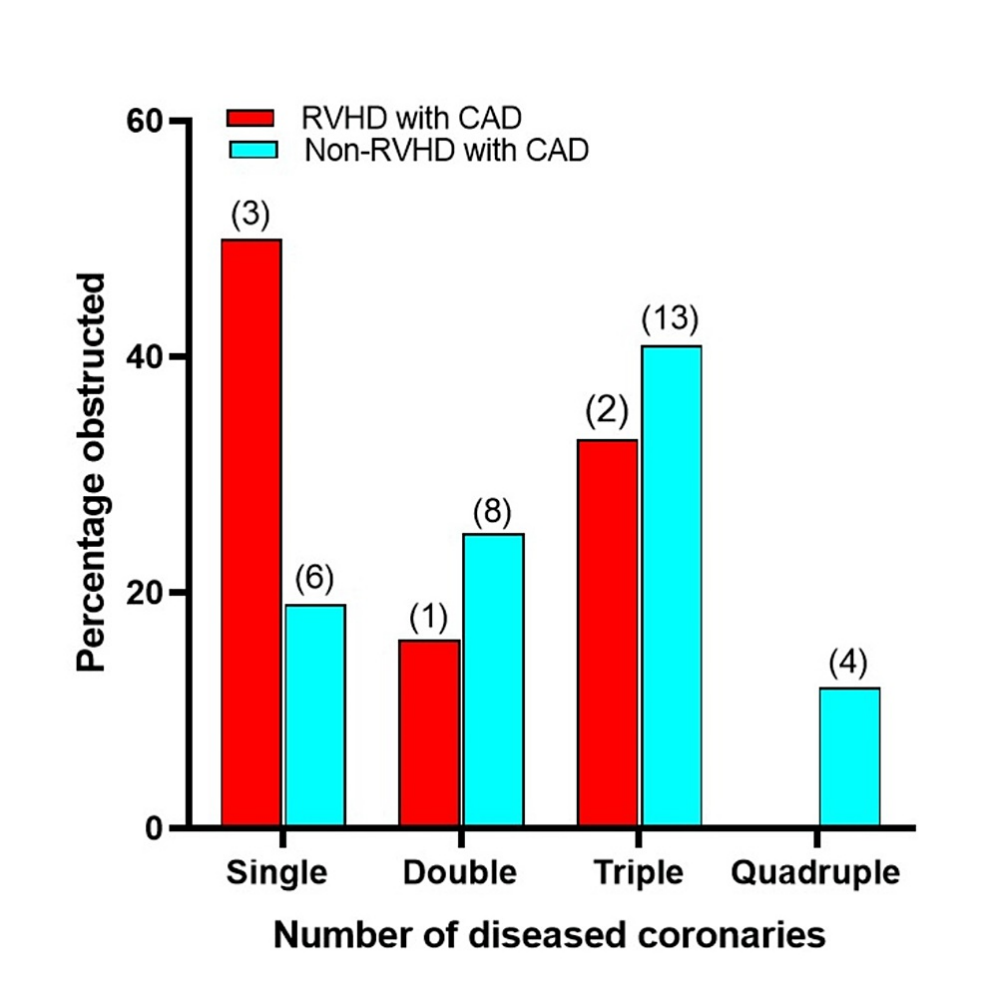
Number of diseased coronaries among rheumatic and non-valvular heart disease patients with coexisting coronary artery disease RVHD: rheumatic valvular heart disease; non-RVHD: non-rheumatic valvular heart disease; CAD: coronary artery disease

Figure 1 shows that in RVHD patients with coexisting CAD (n=6), single-, double-, and triple-vessel disease was seen in 50.0% (n=3), 16.7% (n=1), and 33.3% (n=2) of patients, respectively, while in the non-RVHD patients with CAD (n=31), it was 19.4% (n=6), 25.8% (n=8), 41.9% (13) as well as 12.9% (n=4) had the quadruple-vessel disease.

Table 2 shows that the mitral valve was predominantly affected (100%) in RVHD patients with CAD, followed by the aortic valve (33.3%), and both valves simultaneously to a 33.3% extent, while in the RVHD patients, these were 94%, 62.3%, and 59.5%, respectively. Comparing the RVHD patients (n=37) with non-RVHD (n=32), the involvement of the mitral valve and both valves combined (mitral and aortic) was significantly higher (94.6% vs. 71.9%, ++p ≤ 0.01; 59.5% vs. 31.2% +p ≤ 0.05, respectively).

**Table 2:** Comparison of clinical data (affected valves, diseased valves, and cardiac abnormalities) in rheumatic and non-rheumatic valvular patients with/without coexisting coronary artery disease Fisher’s Exact (value < 5) and Person Chi-square tests were employed for comparisons between groups. Significance of differences: RVHD with CAD vs. RVHD; non-RVHD with CAD vs. non-RVHD, ***P ≤ 0.01, **P < 0.01, *P ≤ 0.05. RVHD with CAD vs. non-RVHD with CAD; +++P ≤ 0.001, ++P ≤ 0.01, +P ≤ 0.032. RVHD: rheumatic valvular heart disease; non-RVHD: non-rheumatic valvular heart disease; CAD: coronary artery disease

Diseased groups	RVHD with CAD	RVHD	Non-RVHD with CAD	Non-RVHD
Total cases (n)	(n=6)	(n=37)	(n=31)	(n=32)
Affected valves	n (%)	n (%)	n (%)	n (%)
Mitral valve	6 (100)	35 (94.6)	23 (74.2)	23 (71.9)^ ++^
Aortic valve	2 (33.3)	23 (62.2)	20 (64.5)	19 (59.3)
Simultaneous both	2 (33.3)	22 (59.5)	14 (45.1)	10 (31.2)^ +^
Valvular heart disease				
Mitral stenosis (MS)	0 (0.0)	8 (21.6)	0 (0.0)	0 (0.0)
Mitral regurgitation (MR)	6 (100)	15 (40.5) **	21 (67.7)	22 (68.7)
Equally MS & MR	0 (0.0)	11 (29.7)	2 (6.4)	0 (0.0)^ +++^
Aortic stenosis (AS)	0 (0.0)	6 (16.2)	15 (48.4)	8 (25.0) *
Aortic regurgitation (AR)	1 (16.7)	15 (40.5)	4 (12.9)	8 (25.0)
Equally (AS & AR)	1 (16.7)	2 (5.4)	1 (3.2)	3 (9.4)
Tricuspid regurgitation	3 (50.0)	17 (45.9)	6 (19.3)	7 (21.9)
Ejection fraction present	6 (100)	5 (13.5) ***	16 (51.6)^ +^	8 (25.0) *
Regional wall motion abnormalities present	3 (50.0)	2 (5.4) **	8 (22.9)	7 (21.2)

No mitral stenosis was detected in both cardiac valvular entities with CAD, and there was no significant difference between the presence of aortic stenosis and aortic regurgitation (Table 2); however, mitral regurgitation was significantly predominant in the RVHD patients with CAD (n=6) compared to the RVHD patients (n=15) (100% vs. 40.5%, **p ≤ 0.01). Aortic stenosis in non-RVHD (n=8) was significantly low compared to the non-RVHD with CAD (n=15) (25.0% vs. 48.4% *p ≤ 0.05).

The cardiac wall abnormalities (ejection fraction and regional wall motion) shown in Table 2 were present in a significantly low percentage of RVHD patients compared to those with coexisting CAD (***p < 0.001; **p < 0.01).

Differences in laboratory variables

Table 3 shows whole blood count, white blood cells (WBC), inflammatory cells (monocytes and neutrophils), neutrophil-to-lymphocyte ratio (NLR) and circulating liver functional markers (alanine aminotransferase (ALT), aspartate aminotransferase (AST), bilirubin and gamma-glutamyl transferase (GGT)) were all significantly lower in the RVHD patients compared to patients with CAD (0.05 ≤ p ≤ 0.001), with exception of albumin level, which was highly elevated (p ≤ 0.001). Overall, these results indicate that RVHD patients with coexisting CAD had a higher prevalence of functional markers. No such significant differences were observed in the non-RVHD patients with coexisting CAD compared to those without CAD. A comparison of coagulation function indexes (prothrombin time (PT), partial thromboplastin time (PTT), and international normalized ratio (INR)) revealed no significant differences between the groups.

**Table 3:** Comparison of laboratory-investigated variables Each value is represented as Mean ± SD using an Independent sample t-test. The significance of differences between RVHD with CAD vs. RVHD and non-RVHD with CAD vs. non-RVHD are indicated by the P values. RVHD and non-RVHD; rheumatic and non-rheumatic valvular heart disease, CAD; coronary artery disease, WBC: white blood cell count; NLR: neutrophil to lymphocyte ratio; ALT: alanine aminotransferase; AST: aspartate aminotransferase; ALP: alkaline phosphatase; GGT: gamma-glutamyl transferase; PT: prothrombin time; PTT: partial thromboplastin time; INR: international normalized ratio; BUN: blood urea nitrogen; TC: total cholesterol; LDL: low-density lipoprotein; HDL: high-density lipoprotein; TG: triglyceride

Variables	RVHD with CAD	RVHD	P-value	Non-RVHD with CAD	Non-RVHD	P-value
Total cases (n)	(n=6)	(n=37)		(n=31)	(n=32)	
Complete Blood Count						
WBC count (x 10^9^/L)	8.84 ± 1.0	6.77 ± 1.9	≤ 0.012	7.35 ± 1.9	7.20 ± 1.7	≥ 0.05
Lymphocytes (%)	27.78 ± 6.1	29.58 ± 11.4	≥ 0.05	31.0 ± 6.5	30.9 ± 9.5	≥ 0.05
Monocytes (%)	6.67 ± 2.0	6.40 ± 1.9	≥ 0.05	6.58 ± 1.9	6.6 ± 1.1	≥ 0.05
Neutrophils (%)	67.5 ± 14.1	60.4 ± 12.6	≥ 0.05	56.8 ± 8.6	56.3 ± 13.0	≥ 0.05
Lymphocytes (x 10^9^/L)	2.34 ± 1.0	1.87 ± 0.50	≥ 0.05	2.12 ± 0.7	2.28 ± 0.7	≥ 0.05
Monocytes (x 10^9^/L)	0.72 ± 0.1	0.52 ± 0.2	≤ 0.01	0.50 ± 0.2	0.52 ± 0.2	≥ 0.05
Neutrophils (x 10^9^/L)	6.28 ± 1.4	4.08 ± 1.4	≤ 0.003	4.69 ± 1.37	4.12 ± 1.5	≥ 0.05
NLR	3.66 ± 2.1	2.39 ± 1.1	≤ 0.05	2.17 ± 2.0	2.14 ± 1.3	≥ 0.05
Liver function						
ALT (IU/L)	23.17 ± 9.1	17.70 ± 5.3	≤ 0.046	17.80 ± 4.9	22.02 ± 13.0	≥ 0.05
AST (U/L)	31.33 ± 10.0	21.98 ± 9.4	≤ 0.032	28.37 ± 20.7	29.81 ± 20.2	≥ 0.05
ALP (U/L)	103.20 ± 51.7	65.87 ± 22.4	≥ 0.05	82.90 ± 27.0	80.90 ± 17.0	≥ 0.05
GGT (IU/L)	66.36 ± 15.4	22.35 ± 11.8	≤ 0.001	30.86 ± 11.1	23.76 ± 9.5	≤ 0.050
Bilirubin (µmol/L)	22.31 ± 1.81	9.70 ± 1.52	≤ 0.020	10.70 ± 1.8	10.10 ± 1.7	≥ 0.05
Albumin (g/L)	34.40 ± 1.5	38.0 ± 4.4	≤ 0.001	36.65 ± 4.3	36.12 ± 5.4	≥ 0.05
Coagulation time						
PT (U/L)	13.80 ± 1.6	14.64 ± 5.1	≥ 0.05	13.22 ± 1.3	13.87 ± 2.4	≥ 0.05
PTT (seconds)	33.40 ± 5.7	34.32 ± 10.0	≥ 0.05	31.87 ± 5.6	34.51 ± 6.0	≥ 0.05
INR	1.08 ± 0.1	0.19 ± 0.31	≥ 0.05	1.12 ± 0.2	1.17 ± 0.2	≥ 0.05
Kidney function						
Creatinine (µmol/L)	90.96 ± 17.0	70.89 ± 15.2	≤ 0.01	87.97 ± 20.6	73.49 ± 18.7	≤ 0 005
BUN (mmol/L)	7.94 ± 3.8	5.05 ± 1.6	≤ 0.003	6.93 ± 2.3	5.33 ± 1.6	≤ 0 002
Calcium (mmol/L)	2.13 ± 0.2	2.09 ± 0.3	≤ 0.044	2.37 ± 0.7	2.02 ± 0.3	≤ 0.025
Potassium (mmol/L)	4.27 ± 0.3	4.26 ± 0.4	≥ 0.05	4.54 ± 0.5	4.25 ± 0.5	≤ 0.037
Lipids						
TC (mmol/L)	3.49 ± 0.7	4.76 ± 0.7	≤ 0.002	3.82 ± 1.0	4.41 ± 0.8	≤ 0.041
LDL (mmol/L)	2.10 ± 0.4	3.34 ± 0.7	≤ 0.001	2.20 ± 0.6	2.90 ± 0.7	≤ 0.001
HDL (mmol/L)	0.79 ± 0.1	1.08 ± 0.2	≤ 0.024	0.87 ± 0.2	0.93 ± 0.3	≥ 0.05
TG (mmol/L)	1.28 ± 0.2	1.39 ± 0.6	≥ 0.05	1.68 ± 0.7	1.47 ± 0.5	≥ 0.05

Levels of creatinine, blood urea nitrogen (BUN), and calcium were significantly lower among RVHD and non-RVHD patients compared to those with CAD (0.005 < p < 0.044) (Table 3). In contrast, the serum total cholesterol (TC) and low-density lipoprotein (LDL) were unexpectedly high in RVHD and non-RVHD patients compared to those with coexisting CAD, except high-density lipoprotein (HDL), which was high only in the RVHD patients.

Predictors of coronary heart disease

Table 4 shows predictor variables and odds ratios for CAD in RVHD and non-RVHD patients based on the results of a bivariate logistic regression analysis.

**Table 4:** Analysis for predictors of coronary artery disease in rheumatic and non-rheumatic valvular heart disease patients with coexisting coronary artery disease as compared to patients without coronary artery disease Bivariate logistic regression analysis was used to calculate the odds ratios (OR) and the variables associated with CAD in RVHD and non-RVHD patients. OR greater indicates a positive relationship. RVHD: rheumatic valvular heart disease; CAD: coronary artery disease; OR: Odds ratio -Exp (β), CI: confidence interval; WBC: white blood count; NLR: neutrophil to lymphocyte ratio; ALT: alanine aminotransferase; AST: aspartate aminotransferase; ALP: alkaline phosphatase; GGT: gamma-glutamyl transferase; PT: prothrombin time; PTT: partial thromboplastin time; INR: international normalized ratio; BUN: blood urea nitrogen; TC: total cholesterol; LDL: low-density lipoprotein

	Bivariate logistic regression			
Predictors of CAD in	RVHD patients		Non-RVHD patients	
RVHD and non-RVHD	OR (95% CI)	P-value	OR (95% CI)	P-value
Hypertension	13.5 (1.4-130.1)	0.024	3.89 (1.30-1.57)	0.015
Dyslipidemia	11.8 (1.2-113.2)	0.032	3.64 (1.25-0.60)	0.018
Diabetes	3.95 (2.06-7.56)	0.001	4.62 (1.60-3.34)	0.005
White blood cells	0.5 (0.3-0.92)	0.026	0.95 (0.71-1.27)	0.735
Monocyte no.	0.001 (0.0-0.41)	0.024	2.22 (0.12-0.48)	0.589
Neutrophil no.	0.39 (0.18-0.84)	0.016	0.75 (0.51-1.10)	0.150
NLR	0.58 (0.3-1.07)	0.080	0.81 (0.57-1.14)	0.229
ALT	0.88 (0.76-1.01)	0.070	1.48 (0.91-2.38)	0.190
AST	0.0 (0.0-204.4)	0.098	1.00 (0.98-1.029)	0.783
GGT	0.85 (0.72-0.98)	0.031	0.96 (0.91-1.00)	0.058
Total bilirubin	0.79 (0.66-0.95)	0.014	0.98 (0.91-1.05)	0.590
Albumin	1.22 (0.98-1.53)	0.075	0.98 (0.88-1.08)	0.671
Creatinine	1.12 (0.51-2.47)	0.760	0.96 (093-0.99)	0.009
BUN	0.65 (0.41-0.99)	0.048	0.66 (0.49-0.89)	0.006
TC	7.39 (1.35-40.49)	0.021	1.94 (1.0-3.75)	0.047
LDL	1.76 (1.20-2.76)	0.003	4.94 (1.71-4.20)	0.003
Ejection fraction	-	-	3.2 (1.10-9.29)	0.032
Abnormal regional wall	17.5 (2.0-149.1)	0.009	3.33 (0.91-2.11)	0.067

Hypertension (OR: 13.5, p ≤ 0.05) and dyslipidaemia (OR: 11.8, p ≤ 0.018) were significantly less in RVHD patients and were more likely to be significant predictors for developing CAD (Table 4). In the non-RVHD patients, hypertension (OR: 3.89, p ≤ 0.015), dyslipidaemia (OR: 3.64, p ≤ 0.018) and diabetes (OR: 4.62, p ≤ 0.005) were also identified as candidate predictors. The WBC, monocytes, neutrophils, GGT, total bilirubin, and BUN levels, were low predictors for developing CAD in RVHD patients (Table 4). In contrast, in the non-RVHD patients, creatinine (OR: 0.96, p ≤ 0.009) and BUN (OR: 0.66 p ≤ 0.006) were the independent factors with a significant negative association. In addition, TC was a strong predictor of CAD in RVHD and non-RVHD patients, whereas, LDL was a very good predictor in the latter group (OR: 4.94, p < 0.003).

Multivariate analysis indicated (Table 5), that BUN, GGT, and creatinine levels were independent predictors of significant CAD development in RVHD patients (Table 5); 84% of the variation in the dependent variable was explained by these risk factors. In the non-RVHD patients BUN, dyslipidaemia, and creatinine retained the threshold significance levels (0.01 < p < 0.05) for a strong association with the risk factors and explained 81% of the changes in the dependent variable.

**Table 5:** Multivariate analysis for the prediction of significant coronary artery disease in rheumatic and non-rheumatic valvular heart disease patients Adjusted OR and (95% CI) in the multivariate analysis were derived from the inclusion of selected significant variables to identify the predictors of CAD in RVHD and non-RVHD patients. RVHD: rheumatic valvular heart disease; CAD: coronary artery disease; OR: Odds ratio -Exp (β); CI: confidence interval; BUN: blood urea nitrogen; GGT: gamma-glutamyl transferase

Multivariate logistic analysis				
	RVHD patients		Non-RVHD patients	
Predictors of CAD in RVHD and non-RVHD	Adjusted OR (95% CI) P-value		Adjusted OR (95% CI) P-value	
BUN	1.57 (1.03-2.52)	0.050	4.81 (1.55-14.91)	0.006
Creatinine	1.12 (1.04-1.20)	0.002	1.07 (1.03-1.23)	0.003
GGT	1.10 (1.01-1.20)	0.016	-	-
Dyslipidemia	-	-	3.39(0.87-13.27)	0.049

## Discussion

There are still somewhat conflicting reports on the prevalence of CAD in patients with RVHD and non-RVHD, even though these three cardiac entities have in common atherosclerosis and several atherosclerotic risk factors [10]. For instance, the overall prevalence of CAD with established RVHD varied between 1.75% and 50% [5,10,19] and in non-RVHD 25% and 40% [10,16-18].

In this study, CAD was found in 13.9% of patients with RVHD and 49.2% with non-RVHD. The prevalence of coexisting CAD in RVHD Saudi patients was significantly lower than in the Western and Pakistani populations [18,20] although comparable to the other population studies (India, China, Nepal) [14,16,21-23]. Other International population studies (India, Brazil, and Spain) reported an even much lower prevalence (1.76-11%) [5,12,13,24]. In non-RVHD patients, CAD was found to be higher compared to studies of others [10,16-18], this may be attributed to the male gender, older age, and ethnicity. The RVHD mainly occurs in children and young adults, whereas non-RVHD occurs typically in older population. However, in this study none of the patients defined angiographically as having CAD were below the age of 60, suggesting that CAD tend to manifest itself at a far later stage in life in the VHD patients.

Single-, double- and triple-vessel disease was common in RVHD and non-RVHD patients with coexisting CAD, thereby reflecting the severity of CAD. However, the pattern of diseased vessels was different. Single- and triple-vessel diseases were high in the RVHD patients with CAD, while in the non-RVHD, triple-vessel disease was high. The findings suggest that the lower prevalence of CAD could be the result of fewer coronaries being affected.

Is the frequency of CAD dictated by the type of valves involved and the pattern of lesions?

Mitral, aortic and double valve involvement was common amongst all patients. However, mitral was involved more than the aortic valve in RVHD patients and those with coexisting CAD, thereby, corroborating the results of others [12,13]. No mitral or aortic stenosis was observed in RVHD patients with coexisting CAD, which explains its low prevalence. In patients with non-RVHD and CAD, both types of stenosis were present, although, aortic stenosis was significantly higher compared to non-RVHD patients, a finding similar to that reported by Matta [17]. This may explain the high prevalence of CAD in non-RVHD patients and the atherosclerosis process in its aetiology. Aortic, mitral and tricuspid regurgitation was a common phenomenon among all patients. Mitral regurgitation was significantly higher in RVHD patients with CAD compared to those without CAD. These results show that mitral regurgitation lesions are more likely to be associated with CAD in RVHD and aortic stenosis with CAD in non-RVHD patients.

Predictors for CAD

In RVHD patients the prevalence of risk factors (hypertension, dyslipidaemia, diabetes), cardiac abnormalities (ejection fraction and regional wall abnormalities), inflammatory markers (WBC, monocytes, neutrophils and NLR), hepatic functional (ALT, AST, GGT, and bilirubin) and renal markers (creatinine, BUN, and calcium) were all significantly lower compared to the patients with coexisting CAD, except for albumin levels. In non-RVHD patients although all these risk determinants were evident, no significant differences were observed, except in the case of GGT and kidney functional markers (creatinine, BUN, calcium and potassium); their prevalence was significantly lower compared to patients with CAD.

In RVHD patients with CAD, the presence of high levels of ALT AST and GGT markers suggests these are associated with the progression of CAD [25,26]. Similarly, high levels of GGT were also noted in non-RVHD patients with CAD.

Bivariate analysis showed that hyperlipidaemia and dyslipidemia were strong predictors of CAD in RVHD, whereas, in non-RVHD patients, this also included diabetes to be a plausibly a good candidate for a predictor of CAD. These associations were analogous to the findings of others [4,10,14,22]. The inflammatory markers (WBC, monocytes and neutrophils), GGT, total bilirubin, and BUN levels were low predictors for developing CAD in RVHD patients. In contrast, in the non-RVHD patients, creatinine and BUN were the independent factors with a significant negative association.

Surprisingly, the TC, LDL and HDL levels were all significantly higher in RVHD patients, whereas the former two lipids were higher in non-RVHD compared to patients with CAD. This was an interesting phenomenon observed because higher lipid levels are known risk factors for CAD. In this study, TC was a strong predictor of CAD in RVHD whereas, in non-RVHD LDL was a very good predictor. It is plausible that high lipid levels, although risk factors of CAD are also involved in the atherosclerosis process of aortic stenosis in RVHD and non-RVHD patients [27].

After the adjustment of all of the significant variables, multivariate logistic regression analysis defined dyslipidaemia and BUN as significant independent risk factors for developing CAD in RVHD patients. In RVHD patients, BUN, creatinine and GGT remained as good predictors of CAD.

Although this study included relatively many VHD patients for a single centre, the number of patients with CAD in non-RVHD was reasonable in numbers, whereas, in RVHD was low. The other limitation of this study was that it had to be terminated due to the COVID-19 crisis.

## Conclusions

There are limited and conflicting data regarding the prevalence of CAD and its predictors. This was the first Saudi patients’ study, which showed a lower prevalence of CAD in patients with RVHD and higher with non-RVHD compared to in Western countries. Risk factors such as inflammatory markers and some liver and renal function markers were significantly low in RVHD, suggesting that in these patients they may have been the attributing factors together with older age, differences in demographics and type of valvular lesions. Further studies are required with a larger sample size and subsets of CAD patients, in this regard to provide the foundation for future strategies for the treatment of CAD and clinical trials.
